# Revealing the mystery of persistent smell loss in Long COVID patients

**DOI:** 10.7150/ijbs.73485

**Published:** 2022-07-15

**Authors:** Jung Woo Park, Xiaoyan Wang, Ren-He Xu

**Affiliations:** 1 Center of Reproduction, Development & Aging, and Institute of Translational Medicine, Faculty of Health Sciences, University of Macau, Taipa, Macau, China.; 2 Ministry of Education Frontiers Science Center for Precision Oncology, University of Macau, Taipa, Macau, China.

**Keywords:** COVID-19, long COVID, olfactory dysfunction

## Abstract

COVID-19 is hopefully approaching its end in many countries as herd immunity develops and weaker strains of SARS-CoV-2 dominate. However, a new concern occurs over the long-term effects of COVID-19, collectively called “Long COVID”, as some symptoms of the nervous system last even after patients recover from COVID-19. This review focuses on studies of anosmia, *i.e.*, impairment of smell, which is the most common sensory defect during the disease course and is caused by olfactory dysfunctions. It remains mysterious how the olfactory functions are affected since the virus can't invade olfactory receptor neurons. We describe several leading hypotheses about the mystery in hope to provide insights into the pathophysiology and treatment strategies for anosmia.

## Introduction

Coronavirus disease-19 (COVID-19) has so far infected more than 400 million and killed nearly 6 million people worldwide since the first reported case in December 2019 1. It is caused by severe acute respiratory syndrome coronavirus-2 (SARS-CoV-2), which infects target cells and hijacks their biological functions 2, 3. The recently emerging variant of SARS-CoV-2, B.1.1.529 (Omicron), is much more transmissible than the previous variants and has spread rapidly in many areas of the world 4. Early estimation indicates that Omicron is less severe than the previous variants, possibly due to its less efficient viral replication 5, 6. Although the morbidity and mortality rates of COVID-19 have not yet slowed down, many countries are preparing to undertake COVID-19 as an endemic disease and slowly crawling back to normalcy through natural and vaccine-induced immunity 7.

However, what remains to be a concern regarding COVID-19 is the persistent symptoms, ranging from fatigue, headache, shortness of breath, smell and taste loss, and depression to psychiatric and cognition defects, which continue to affect millions in their daily life 8-11. These symptoms, collectively called “Long COVID”, typically last more than 12 weeks and even longer than one year from the onset of the disease. A recent systematic review reported that more than 50% of the COVID-19 survivors suffer at least one of the post-acute sequelae of COVID-19 (PASC) or long COVID symptoms 12, 13. One prevailing thought for long COVID is the viral persistence in COVID-19 patients, even after the recovery from the disease. Persistent SARS-CoV-2 infection found in certain cohorts with immune deficiency may be a major contributing factor to the persistent symptoms 14.

One of the most common symptoms associated with COVID-19 is the loss of smell, which was previously unrecognized in other CoV-related diseases. Multiple meta-analyses indicate that more than 50% of COVID-19 patients suffer from olfactory impairment during an early stage of the disease or even months after the recovery 15-17. A study using a microencapsulation assay named University of Pennsylvania Smell Identification TEST reported higher prevalence of olfactory dysfunction (OD) than other studies did based on self-reports such as questionnaires and interviews 18. The olfactory function is evolutionarily conserved as one of the oldest senses, given its importance in identifying food, mating partners, and escaping dangers 19. Considering the physiological importance of olfaction and widespread OD prevalence among COVID-19 patients, here we summarize studies on the pathogenesis of OD manifested in COVID-19 and discuss the potential mechanisms of persistent OD as well as treatment options.

## Olfactory dysfunctions associated with COVID-19

Intriguingly, many COVID-19 patients and survivors reported central nervous system (CNS) symptoms, including headache, olfactory and taste dysfunctions, seizure, stroke, and even long-term cognitive dysfunctions 11. Several studies have detected both RNA and protein of SARS-CoV-2 in the brain tissues from COVID-19 autopsies and animal models. The manifested neuro-invasion by SARS-CoV-2 implicates that the virus enters through damaged endothelial cells of the blood-brain barrier (BBB), lymphatic systems associated with respiratory organs, infected immune cells, and neural-mucosal interface 20, 21. Since the virus enters the brain via the neural-mucosal interface, OD is considered as an important diagnostic and prognostic indicator of the long-term neurological complications associated with COVID-19.

At least 40% of COVID-19 patients reported anosmia as an early sign of the disease, and nearly 70% of patients with OD claimed a reduced quality of life due to smell loss 13, 22. More than 10% of the infected with COVID-19 may suffer prolonged anosmia, often more than one year from the onset of the disease 17, 23. A similar but distinct symptom of the OD, called parosmia, described as a distorted or unmatched smell, was found to be less common than anosmia during the duration of COVID-19 but substantially higher in the follow-up surveys conducted months later 24. This suggests that parosmia could be a valuable diagnostic marker for long COVID.

## Epidemiological analysis of COVID-19 associated OD

Many meta-analyses have been carried out to study OD frequency concerning age, sex, potential risk factors such as smoking, and genetic associations. However, variable and even contradictory results were reported in associating OD with the aforementioned factors among COVID-19 patients 15. This could be mainly due to the experimental variables of the study methods, design, and sample number. For example, most reports on olfactory defects due to COVID-19 are based on self-reports, questionnaires, and other subjective criteria that may hinder the objectivity of the studies.

Despite the inconclusive premises of the studies, several reports provided meaningful epidemiological observations associated with OD. First, smell loss was observed more commonly in asymptomatic COVID-19 cases than severe cases. A study demonstrates that patients with OD suffered less severely in terms of hospitalization and mortality rate 13. Second, age is considered a major risk factor in COVID-19 related OD 25. For instance, the expression of endogenous angiotensin-converting enzyme 2 (ACE2), the binding receptor of SARS-CoV-2 on target cells, increase with age in mice, suggestive of the age-dependent OD prevalence and severity 26. One caveat of the studies is that there is a general decline of the olfaction in later life, which may lead to overestimation of the extent of smell loss. One large-cohort study involving more than 70,000 individuals who reported symptoms found an inverse correlation between age and the frequency of smell loss 27. Third, several reports suggest no significant association between sex and OD regarding the prevalence and severity 18, 28. It is interesting to note that a recent study examined the expression profiles of the obligate receptor ACE2 and the accessory protease transmembrane serine protease 2 (TMPRSS2) in thousands of cells from hundreds of biopsy samples from different individuals using single-cell RNA sequencing (scRNA-seq) 29. The study indicates a strong correlation between sex, age, smoking, and expression of ACE2 and TMPRSS2, suggesting a higher prevalence of OD in males, elders, and smokers than in females, youngers, and non-smokers, respectively 29.

## Structure and function of Olfactory system

### Anatomy, organization, and physiology of the olfactory mucosa (OM)

The OM is in the roof of the nasal cavity and detects odorants as they enter the nasal cavity. It is a mucus-secreting membrane structure composed of the olfactory epithelium (OE) and lamina propria (LP) beneath. The OE houses multiple cell types, including olfactory receptor neurons (ORNs), also known as olfactory sensory neurons (OSNs), and many supporting/glial cells. ORNs are bipolar neurons with dendritic ends containing multiple protruding cilia which project into the mucosal cavity. The unmyelinated axon bundles extend through the cribriform plate to form a synapsis with mitral and tufted cells in the olfactory glomerulus within the olfactory bulb (OB). Subsequently, the synaptic signal is transmitted to the projection interneurons and ultimately to the primary olfactory cortex and anterior hippocampus for smell recognition and memory retrieval 30, 31.

Besides ORNs, the OE is composed of sustentacular cells (SCs), Bowman's gland ductal cells (BGDCs), horizontal and globose basal stem cells (HBCs and GBCs), and microvilli cells (MVCs) 32. SCs are morphologically epithelial cells that enwrap the dendrites of the ORNs and mediate the functions of the ORNs by metabolic, secretory, and phagocytic activities 33, 34. The primary function of BGDCs is secretion of fluids, including mucin, which forms the mucus layer covering the OE. HBC and GBC are multipotent stem cells located at the basal layer of the OE. They can differentiate into both neuronal and non-neuronal cell types to maintain the homeostasis of the OE 35. MVCs are morphologically similar to SCs and send out microvillar toward the surface of the olfactory mucus, however, the precise function of MVCs is not yet known 36.

### Chemosensory mechanism of the olfactory system

The chemosensory activation of the ORNs has been described in detail in the following reviews 37, 38. Briefly, odorants reach the OE within the olfactory cavity; an odorant binds to a specific odorant receptor (OR), which belongs to the G protein-coupled receptor (GPCR) expressed in the cilia of the ORN. The OR bound by odorants activates a cAMP-based second messenger cascade and then the G protein, G_olf_, which further activates adenylyl cyclase III and increases cAMP level in the cilia. The subsequent opening of ORN channel triggers the Ca^2+^ influx and opens the chloride channel, leading to the depolarization of the ORN. The axons of the ORNs expressing a specific receptor and propagating an action potential migrate through the cribriform plate and join other ORNs to form fascicles, innervating glomeruli in the OB.

### ORNs and smell recognition

ORN genes constitute the most extensive gene family in the mammalian genome 39. The dimension and diversity of ORNs genes in the animal genome may be attributable to the need to detect and discriminate the distinct and diverse odorants 31. The current hypothesis, known as the one neuron-one receptor hypothesis, describes that an ORN transmitting the signal for a unique and specific smell cue is facilitated by the monoallelic expression of a single OR in a mutually exclusive manner 40, 41. Multiple mechanisms, including DNA recombination, gene conversion, RNA decay, and activation by the locus control region (LCR), have been implicated in achieving the single OR expression 41, 42. Moreover, it has been demonstrated that the expression of an OR per ORN is critical to not only transmit a specific signal for a unique odorant detection but also to facilitate the axon guidance and correct targeting of the ORN to a specific glomerulus 43.

However, unlike other sensory neurons, the olfactory system exhibits an extraordinarily complex organization to recognize a broad spectrum of odorant molecules and integrate the signals to transmit to the olfactory cortex. A recent study carefully analyzed the OR expressions through scRNA-seq of individual ORNs during olfactory development in *Drosophila melanogaster*, which revealed two critical principles 44. First, the same set of receptor genes is expressed continuously in any given ORN during development. Second, the transcriptomic profiles are distinctively maintained in anatomically and functionally defined ORNs throughout the developmental stages. Thus, the OE is spatially organized and ORNs in any defined area may be associated with sensing of a specific group of odorant molecules.

## The OM as key entry target for SARS-CoV-2

The mechanism by which SARS-CoV-2 enters the host cells has been discussed in recent reviews 45, 46. Briefly, the trimeric form of the glycoprotein, Spike (S), encoded by the 3,822-bp S gene, binds to ACE2 and undergoes the cleavage catalyzed by TMPRSS2. Subsequently, the activated Spike protein fuses with the host cell membrane, followed by endocytosis of the viral particle. Given the location of the OM in the nasal cavity, the viral load in the OM is among the highest in the comparative analysis of the swab species from multiple organs, including bronchioles, pharynx, sputum, blood, and urine 47, 48. More importantly, many cell types constituting the OE express both ACE2 and TMPRSS2 49. In fact, Hou *et al.,* revealed the infection gradient based on the ACE2 expression level of various body tissues 50. The viral titer was the highest in the nasal cavity, where ACE2 expression was the lowest in the alveoli of the respiratory tract 50. Therefore, it is likely that the OM is the initial infection site for SARS-CoV-2 and manifests olfactory loss as one of the earliest symptoms of COVID-19. Finally, consistent with the notion that D614G mutation rapidly accelerated the viral SARS-CoV-2 transmission by increasing affinity between the receptor binding domain of S protein and ACE2, the G614 variant causes a statistically higher frequency (~6-fold) of anosmia than the D614 variant, further suggesting that the OD is a key diagnostic marker for COVID-19 pathogenesis 51.

## Genetic factors as a determinant for COVID-19 associated OD

Whereas OD is affected by many factors in the nasal microenvironment including the viral titer, the pre-existing inflammatory condition or immune-deficiency 52, it may also be associated with the genotype of individuals, *i.e.*, genetic factors may determine the prevalence and severity of COVID-19 associated OD.

First, East Asian populations displayed a 2-3-fold lower rate of olfactory and taste defects than the populations in western countries 53. The higher susceptibility to the OD was not due to the disproportionate SARS-CoV-2 variant types or mutation rates found in different geographical locations. Instead, the differential expression of ACE2 due to polymorphism found in different populations were found to contribute to differential susceptibility 54, 55. In particular, the allele frequency of an intronic variant SNP rs2285666 (G8790A) in Asians is nearly 2-fold higher than the other populations, and is associated with lower SARS-CoV-2 infection 56. Another study indicated a higher concordance and correlation in anosmia among monozygotic twins than dizygotic twins, suggesting a correlation between genetics and the COVID-19-caused anosmia 57. However, a study involving 1,700 variants from a genome database analyzed the polymorphisms in the coding region of ACE2 in different populations and found no direct correlation among the genetic polymorphism, susceptibility, and symptoms of COVID-19 58.

Shelton *et al.,* recently conducted a genome-wide association study among more than 69,000 self-reported COVID-19 patients 27, and identified the link of a locus at chr4q13.3 containing *UGT2A1* and *UGT2A2* to the smell loss 27. This is the first study linking COVID-19-caused olfactory defects to genetic polymorphisms in large populations. Although the cellular mechanism related to this locus is unknown, drosophila homologs for *UGT1A1* and *UGT2A2* are known to play an important role in olfactory functions. Reduced detection of pheromone and other functional defects were observed with tissue-specific mutations and RNAi-mediated knockdown of *UGT1A1* and *UGT2A2* 59.

## Discrepancy between the expression pattern of ACE2 and SARS-CoV-2 infection

**Figure 1:**
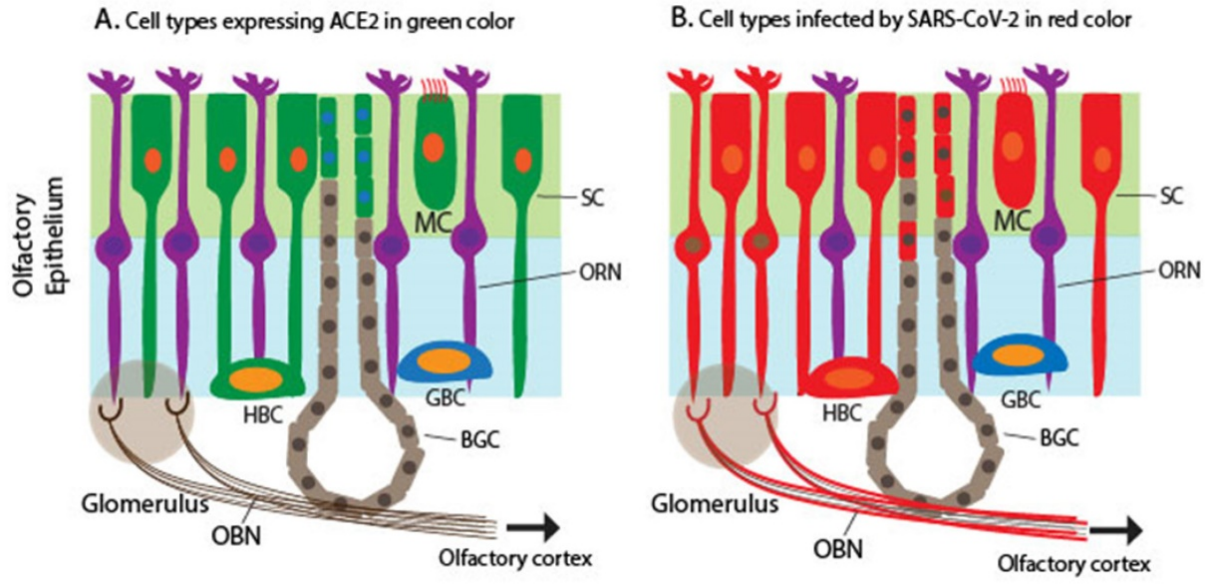
** Schematic to illustrate the expression of *ACE2* encoding a receptor for SARS-CoV-2 and detection of SARS-CoV-2 in the olfactory mucosa.** A. Cells expressing *ACE2* in the olfactory mucosa, which are labeled in green, including SCs, MCs, a subset of BGDCs, and HBCs. B. Cells infected by SARS-CoV-2 in the olfactory mucosa, which are labeled in red, including SCs, MCs, HBCs, BGDCs, a subset of ORNs, and cells in the outer layers of OB (the mitral and glomerular neurons), based on the detection of the viral RNA and antigens.

SARS-CoV-2 enters the host cells through its binding receptor ACE2 and protease TMPRSS2. Several scRNA-seq studies determined the expression pattern of *ACE2* and *TMPRSS2* in the nasal mucosa, particularly ciliated apical OE in humans, non-human primates, and mice 49, 60-63.* TMPRSS2* expression is ubiquitous in many cell types, including neuronal and non-neuronal cell types in the OM, although TMPRSS2 expression is higher in non-neuronal OE cells than in ORNs 49, 64. A single cell meta-analysis of hundreds of tissue types from more than 200 human subjects demonstrates that the OM shares the expression programs, including *ACE2*/*TMPRSS2* co-expression, with other SARS-CoV-2 target cells such as a subset of alveoli cells and airway secretory cells 29. Together, *ACE2* expression is considered the most significant susceptibility factor for viral entry. Multiple studies have shown that ACE2 and TMPRSS2 expression is restricted to SCs, MVCs, and BGDCs in the OE of humans and mice 60, 61, 65 (Figure 1A). As mentioned above, SCs are non-neuronal columnar epithelial cells and play a critical role in enwrapping ORNs and other supporting functions such as the nutrient exchange and removal of dead debris and neurons. SARS-CoV-2 infects mouse SCs that express human ACE2, leading to massive cell death of the OE, immune cell infiltration, and subsequent damage to the OE structure 65.

However, Meinhardt *et al.,* recently documented the detection of S protein in the cytoplasm and perinuclear regions of two cell types: cells with epithelial morphology and dendrite-harboring cells in the OE 21. This indicates that SARS-CoV-2 appears to infect both non-neuronal and neuronal cell types in the OE of the OM. The infection of ORNs is confirmed by the co-localization of S protein in a subset of TuJ1/NF200/OMP+ ORNs 21. The finding was confirmed through detection of viral RNA in both ACE2-expressing non-neuronal cells and non-ACE2-expressing neuronal cells as well as in CNS tissues derived from COVID-19 autopsies 21, indicating that the virus invaded non-ACE2 expressing neurons including ORNs. Other studies have also shown various levels of SARS-CoV-2 proteins and RNA in human samples 60, 61, 66, 67(Figure 1B). Furthermore, animal studies, including mice, hamsters, and ferrets, produced similar results in that SARS-CoV-2 proteins or RNA were detected in non-ACE2 expressing cells 65, 68, 69. The reason for the discrepancy between the ACE2 expression profile and SARS-CoV-2 infection pattern is unknown.

Most studies indicate that the infection of neuronal cell types not expressing *ACE2* is a rare event, as indicated by the extremely low percentage of the co-localization between SARS-CoV-2 antigen and neural markers, particularly ORN markers. What are the possible routes of viral entry to ORNs? One possible mechanism by which the ORNs are infected is through the differentiation of the infected HBCs since HBCs have been shown to express low levels of *ACE2* and *TMPRSS2* 70. Once the ORNs are infected, the virus could transverse along the ORN axons and infects the post-synaptic neurons through an uptake using the neurotransmitter system, further infecting olfactory bulb neurons (OBNs) and interneuron *en route* to the olfactory cortex. It has been shown that herpes simplex virus type I infects the CNS through this route by initially infecting OBNs 71. Alternatively, ectopic *ACE2* expression within a subset of neurons may result in the viral tropism of the neuronal cell types. Other cell-cell communication mechanisms such as the secretion and absorption of the extracellular vesicles (EV) and the transmission of the virus through the gap junction cannot be excluded 72, 73.

For neurons that don't express ACE2, they may be infected through induced expression of ACE2 following interferon (IFN) signaling. SARS-CoV-2 dsRNA induces the activation of type I IFN molecule in ACE2-expressing cells in the OE. Subsequent secretion of IFN-α activates type I IFN signaling and a series of IFN-stimulated genes (ISGs). The increased expression of ISGs provides anti-viral activities by stimulating innate immune cells 74. Ziegler *et al.,* demonstrated *in vivo* and *in vitro* that both type I and II IFN signaling could induce *ACE2* expression in both nasal and respiratory epithelium 63. This suggests that IFN-α and IFN-γ secreted from SCs and the infiltrating immune cells can induce *ACE2* expression in the neighboring cells, including ORNs 63. Interestingly, it was also shown that the non-structural proteins, *i.e.*, nsp1 and nsp13 of SARS-CoV-2 could directly inhibit type I and II IFN signaling by blocking the phosphorylation of STAT1 and STAT2, indicating that there exist intimate and more complex cross-talks between the viral tropism and immune response 75, 76. Activation of IFN signaling by SARS-CoV-2 infection, in turn, can induce *ACE2* expression and exacerbate the infectivity of SARS-CoV-2. This gives the upper hand to the virus in infected cells that generally do not express *ACE2*.

## Pathogenesis of OD caused by SARS-CoV-2

The temporary smell loss can be attributable to three primary sources of dysfunction in the olfactory system. First, the blockage of the nasal passage and mucosal tissue due to local inflammation can prevent odorant molecules from reaching the OE in the roof of the nasal cavity. The level of pro-inflammatory cytokines produced by the infected cells in nasal mucosa has been considered a strong indicator of the disease severity 77. Second, the temporary olfactory dysfunction can be caused by an altered level or function of ORNs. Third, temporary functional defects in the signal processing parts of the olfactory system, *i.e.*, olfactory bulbs and the olfactory cortex, can contribute to transient smell impairment.

In a hamster model, nasally applied SARS-CoV-2 massively damaged the OE as early as 2 days post-infection (dpi), demonstrated by the reduced thickness of the septum OE and shedding of the ORN cilia into the lumen 68. The severity of the damage exacerbates 4 dpi and gradually decreases by 14 dpi. However, both the OE thickness and damage levels do not fully return to the normal levels 68. Significantly, the cilia of the ORN lose as much as 90% of G_olf_ protein detection in the ORN. The most severely affected cell type appears to be SC, as the majority of SCs are positive per immunostaining for SC-specific marker Keratin-18 (K18) and the SARS-CoV-2 N protein. Finally, increased macrophage and monocyte infiltration levels were observed in the OE and the *lamina propria* of the infected animals. Together, both the severely damaged SCs and infiltration of immune cells affect the integrity and functions of ORNs.

Most COVID-19 survivors with OD symptoms recover in a few weeks, whereas others continue to suffer anosmia for months after its onset 78. For COVID-19 patients with transient anosmia, damaged cells in the OE are replenished by activation of HBCs. In contrast, chronic or persistent anosmia may be caused by the persistent presence of the virus in the OM of the patients, leading to chronic inflammation in many cell types, including ORNs 66. In addition to prolonged inflammation, other mechanisms, *i.e.*, cell-autonomous and non-cell-autonomous mechanisms may cause chronic or permanent dysfunction in the olfactory system and will be described below in detail.

## Role of the olfactory pathway in SARS-CoV-2 entry to the CNS

Findings on autopsy samples from COVID-19 victims have revealed that SARS-CoV-2 virions invade the defined neural regions, including OB, trigeminal ganglions, and medulla oblongata, as determined per RT-PCR, *in situ* hybridization, and histocytochemistry 21. Consistent with the viral penetration of the virus to the CNS, the infiltration of macrophages and CD8^+^ T lymphocytes in perivascular regions and widespread microglial activation throughout the brain were observed, indicating virus-induced neuroinflammation 21. Others have also demonstrated the infection of brain tissues in both humans and mice by SARS-CoV-2, whereas the studies didn't propose a possible entry route to the brain 79, 80.

Recent studies have implicated multiple routes and mechanisms by which SARS-CoV-2 invades the CNS and brain. It has been suggested that the virus may directly enter the CNS through the brain capillary endothelial-like cells (BCECs) within the blood-brain-barrier (BBB) structure 81. Analysis of cerebral-spinal fluid shows up-regulation in the expression of interferon-regulated genes in dendritic cells, along with activated T cells and natural killer (NK) cells. This is accompanied by increased interleukin-1 (IL-1) and IL-12, not seen in the blood plasma 82. Another suggested mechanism involves neuropilin-1 (NRP1), a transmembrane glycoprotein with a capacity of binding to the furin-cleaved substrates. Cleaved S protein by furin or TMPRSS2 can activate NRP1 for the viral entry into NRP1-expressing endothelial and epithelial cells 83. Meinhardt *et al.,* proposed that the virus invades the CNS through the axons in the ORNs, which project to specific neuronal areas 21. The presence of intact SARS-CoV-2 particles in ORNs, neuroanatomical regions, and olfactory track projections suggests neural invasion through axonal transportation 21. More studies are needed to further define the mechanisms whereby SARS-CoV-2 invades the CNS.

## Pathologic mechanism for COVID-19-associated persistent smell loss

Although the temporary smell loss caused by COVID-19 often returns to normal within a few weeks after the recovery from COVID-19, persistent olfactory loss can be a long complication affecting the life quality, for which little is known about the mechanisms. Based on the current literature, we discuss two leading and contrasting views on the cause of persistent anosmia.

### Cell-autonomous mechanism

Analyses of olfactory tissues from COVID-19 autopsies indicated viral infection in the olfactory bulb (OB), based on the detection of the viral RNA and protein 21, 84, 85. In addition, histological and neuroimaging analyses showed a high level of OB damage and both CD148 (an inflammatory marker) and viral antigen were detected in the outer layers of the OB isolated from COVID-19 autopsies 33, 80, 86. As *ACE2* expression is widely distributed in the glomerular and mitral layers within the OB, it is possible that SARS-CoV-2 infects the outer layers of the OB and triggers TLR4-mediated inflammatory response 49, 87.

**Figure 2:**
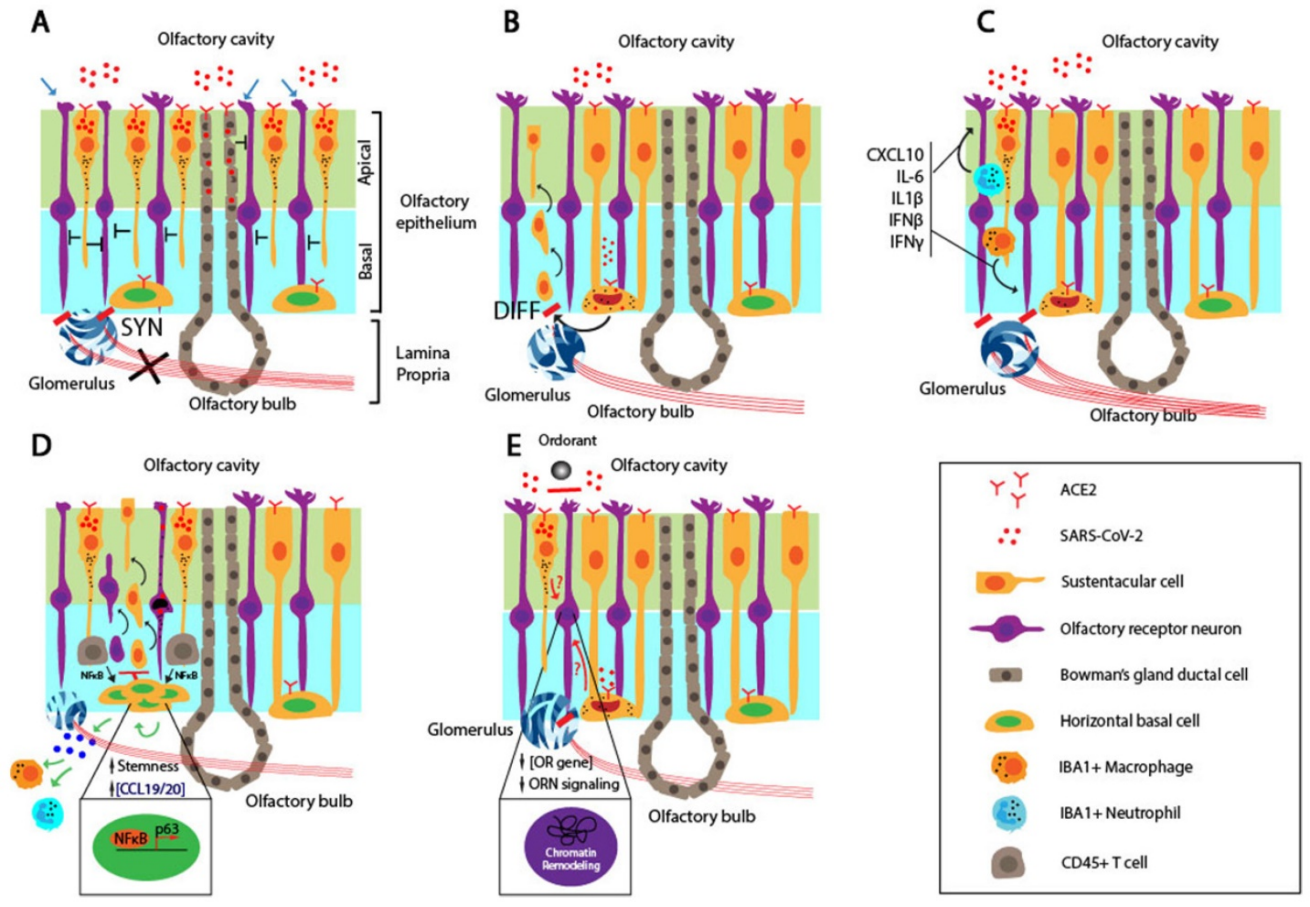
** Schematic to illustrate mechanisms for persistent OD associated with COVID-19.** A. Infection of the mitral and glomerular cells by SARS-CoV-2 through ACE2-mediated cell entry and low neurogenesis potential of OBNs manifested in permanent smell loss. B. Massive damage and cell death of SCs and ciliated apical side of the OE by SARS-CoV-2 invading through ACE2, illustrated as “Y” shape. The extensive damage to the OE due to the SC cell death affects the ORN structures and functions, leading to permanent smell loss. C. Infiltrated IBA^+^ neutrophils and macrophages produce inflammatory cytokines, including CXCL10, IL-6, IL-1β, IFNβ, and IFNγ, and affects ORN functions. The persistent presence of the virus results in the prolonged inhibitory activity against the ORNs. D. SARS-CoV-2 infection of the HBCs disrupts ORN differentiation and maturation as well as the restoration of the ORN after the OE damage. Ultimately, the ORN functions are inhibited or damaged for an extended period. E. The exposure to TNFα triggers differential NFκB signaling in the HBCs. The early or chronic inflammation in the OE promotes differentiation and proliferation of HBCs by upregulating the expression of differentiation genes and *p63*, respectively. imORN and mORN stand for immature and mature ORNs, respectively. F. SARS-CoV-2 infection of SC causes chromatin re-organization such that the OR cluster no longer interacts with the enhancer sequence, thereby downregulating the expressions of OR and ORN signaling genes and disrupting the functions of ORN in the long term. The red dots, black dots, and the half-moon or crescent-moon-shaped objects inside the cells represent SARS-CoV-2, apoptotic granules, and dying nuclei, respectively.

It is estimated that OBNs undergo less than 1% turnover rate over 100 years based on ^14^C levels in genomic DNA in the human olfactory system 88. OBNs are formed by the subsequent migration and maturation of the neuroblasts, differentiated from multipotent neural stem cells that originate from the subventricular zone in the cerebral cortex 89. Thus, it is challenging to replenish OBNs following their damage or death due to the low neurogenesis potential in adult humans (Figure 2A).

Several animal models showed that SARS-CoV-2 infects, and causes massive damage to, the apical ciliated OE structure, and neutrophils and macrophages recruited to the OE further damage the OE 65, 66, 90. Regardless of the ACE2 expression pattern, the primary targets for SARS-CoV-2 are SCs, MVCs, and BGDCs, but not ORNS located in the OE 60, 91. Extensive damage to SCs in the OE leads to functional and structural loss of the OE despite the regenerative potential of stem cells residing in the basal layer of the OE after the injury. It is generally thought that both ORNs and supporting cells are continuously regenerated from stem cells within the OE after the injury. However, the persistent infection by virus and inflammatory microenvironment within the OE may undermine the rate of the regeneration potential and delay restoration of the OD even weeks after the recovery (Figure 2B). This may occur in conjunction with the death of OBNs, resulting in permanent smell loss.

SARS-CoV-2 infection of the OE results in an inflammatory environment. A recent report by Ho et al. provided evidence for the degeneration of olfactory axon injury microvasculopathy in the post-mortem samples, and this indicates a local inflammation in the micro-vessels 92. It is accompanied by the infiltration of the innate immune cells, as evidenced by co-staining for IBA, a myeloid marker, and the N protein of SARS-CoV-2 66. Myeloid-derived macrophages and neutrophils recruited to the infected OE secret pro-inflammatory cytokines. Moreover, biopsy samples from COVID-19 patients with persistent smell loss showed high levels of IBA+ immune cells and up-regulated expression of IL6 in the olfactory mucosa 66. It is interesting to note that a high level of the viral genomic RNA, but not subgenomic RNA, was present in the olfactory mucosa, indicating no active viral replication in these cells 66. Together, these findings suggest that the persistent inflammation in the OE affects the ORN and OBN functions (Figure 2C). The persistent presence of the genomic RNA of SARS-CoV-2 may induce IFN expression in an RNA-dependent and TLR-mediated manner, trigger ACE2 expression in neighboring cells, and cause their reinfection by the virus and infection of cells that don't express ACE2 93, 94.

Brann *et al.,* showed that most of the HBCs express the required receptors, ACE2 and TMPRSS2, which favors the entry of the SARS-CoV-2 60. KRT5-positive HBCs derived from biopsy samples of a COVID-19 patient were co-stained positive for ACE2 in the cell body 60. Furthermore, the level of ACE2 is elevated upon the activation of HBCs, compared to the resting HBCs. In a murine model, the nucleocapsid (N) protein of the SARS-CoV-2 was detected in CK8^+^ SCs, CK8^+^/Sox9^+^ BGCs, and CK5^+^ HBCs. The SARS-CoV-2 infection of HBCs might cause persistent OD due to reduced regeneration capacity of the OE in a long term (Figure 2D). In cell-autonomous models, the OD directly involves the infection, followed by cell death or functional loss of the neuronal components, *i.e.*, ORNs and OBNs, coupled with delayed or reduced regenerative capacity of the OE stem cells.

### Non-cell-autonomous mechanism

Chronic rhinosinusitis (CRS) is a common chronic inflammatory disease of the airway caused by various conditions such as infection and dysfunctions of the sinus. Although its exact etiology is unknown, it has been shown that the infiltration of local immune cells and production of inflammatory cytokines cause the loss of ORNs and reduced olfactory function 95. Anosmia associated with COVID-19 can be described in the context of CRS. While studying the cause of the smell impairment in CRS, Chen *et al.,* identified a novel mechanism in which the prolonged inflammation locks the HBCs in an undifferentiated state, in part, by upregulating p63 96. The inflammation at the early stage of COVD-19 results in production of pro-inflammatory cytokines such as TNFα. It induces HBC to differentiate to ORNs in an NFκB-dependent manner. However, the persistent NFκB signaling lowers the differentiation potential of HBC by upregulating transcription factors involved in maintaining the stemness of HBC (Figure 2E). In this model, the infection or dysfunction of ORNs is not a prerequisite for persistent smell loss.

A recent study by Zazhytska *et al.,* raised an intriguing idea in which SARS-CoV-2 suppresses the ORN functions without infecting the ORNs 67. Right after the SARS-CoV-2 infection of golden hamsters, the authors detected the viral infection and massive death of the SCs. However, the ORN and OBN infection level was extremely scarce, and these cells remained intact. After transcriptomic analysis of all cells derived from the OE via scRNA-seq, the authors discovered the striking downregulation of ORs and ORN signaling genes in the ORNs. Furthermore, the nuclear chromatin structure was massively reorganized in the ORNs of the infected animals compared to mock controls. Using *in situ* HiC to measure and quantify the pairwise interactions between two chromosome regions, the authors revealed that physical interactions between OR gene clusters in cis- and trans-contacts were drastically reduced in the infected animals.

Interestingly, the serum depleted of cells by UV from infected hamsters induced a global disruption of the nuclear chromatin structure in ORNs in a non-cell-autonomous manner, which indicates that the soluble factors in the serum of the infected animals may induce the reorganization of the chromatin. The identity and origin of the soluble factors are not known. Nonetheless, the viral infection may induce secretion of the soluble factors, remodel the chromatin within the OR gene clusters, and downregulate the expression of ORs and corresponding signaling genes in the ORNs 67 (Figure 2F).

It has been recently suggested that the SARS-CoV-2 genome can integrate into the genome of host cells in a LINE-1 dependent manner 97. The genomic RNA of SARS-CoV-2 is integrated into the genome of the cultured lung cells or organoids after the viral infection. The integration was evidenced by the chimeric reads spanning human-negative-strand RNA of SARS-CoV-2 were found in cells derived from deceased COVID-19 patients 97. It is interesting to note that the detected RNA in the infected ORNs was genomic, but not subgenomic, RNA, indicating that the integrated viral RNAs are non-replicating in nature 66. Nevertheless, the hypothesis or conclusion that the genomic RNA of SARS-CoV-2 integrates into the human genome met abundant critiques as currently, there is no plausible mechanism for the negative-strand RNA of SARS-CoV-2 to integrate into the human genome. Nonetheless, this phenomenon could be in line with the activation of *IFN* and *ISGs* by the viral RNA, leading to persistent infection and damage to the OE, hence permanent OD. Together, non-cell-autonomous models depict the transcriptomic changes in HBCs and chromatin remodeling in ORNs without SARS-CoV-2 infection as the primary causes of persistent OD.

## Potential stem cell therapy for persistent OD

While most COVID-19 patients with the symptoms of OD recover from the smell loss, as many as 1.6 million people in the U.S. alone may suffer from permanent smell loss 98.

The current mainstream therapy for chronic or permanent olfactory dysfunctions include smell training, oral or topical steroids, and nonsteroidal oral medications 99-102. Based on the available evidence, smell training is a recommendation with minimal harm effect and highest benefit in improving olfactory function. The only inconvenience is the need for a sustained daily training for months. The use of short-term oral and/or topical steroids is an option, especially for their anti-inflammatory activity. Considering the potential side effects relating to systemic corticosteroids, the patients should be carefully selected. Numerous nonsteroidal oral medications also show their values in relieving the symptoms of OD, especially when they benefit from wide accessibility and high tolerance. However, more rigorous evaluations are required when contradictory and inconsistent results are produced 103. Recently, Platelet-rich plasma (PRP) therapy has been applied to treat anosmia patients in the US with a potential promise 104. PRP therapy has been utilizing the injection of one's own plasma containing platelets to promote the healing of injured ligaments, joints, and muscles. For long term or permanent smell loss, the function of the olfactory neurons is often affected, with neurogenic exhaustion as the common feature. Stem cell-based therapy may be broadly effective in replacing the damaged or senescent ORNs. Given the current understanding of the olfactory and neural stem cells, they are potential sources for cell therapy of permanent OD to restore olfactory functions.

### Stem cells in the olfactory mucosa

The OM is directly exposed to the external environment, thus vulnerable to insults including chemicals, viruses, and bacteria, which can cause neuronal cell damage and death. The presence of stem cells in OM maintains the homeostasis of the olfactory cells and functions. The pseudostratified OM consists of mature ORNs, supporting cells, and two distinct populations of basal cells. The two basal cell types are HBCs in flat morphology, positive for Keratin-5 (K5), and the most basally located cells in the OE and GBCs, which lie above the HBC layer. Unlike other parts of the nervous system, the OE retains the capacity for neurogenesis and maintains the olfactory sense during a lifetime 105. It was thought that HBCs remain quiescent during a normal homeostatic state, and GBCs are sufficient to replenish the whole OE 106. HBCs become active, morphologically changing from a flattened to pyramidal shape, and regenerate the entire OE when there is substantial damage to the OE 106, 107. However, a fate-mapping study by Carter *et al.,* demonstrated that HBCs are quiescent and multipotent and can differentiate into both neuronal, *i.e.*, ORNs, and non-neuronal lineages, including SCs and BGDCs, even during the normal turnover of the OE 35. Moreover, GBCs were derived from HBCs, indicating that HBCs give rise to all cell types, including GBCs in the OE 106. It is important to note that p63, a transcription factor, is a master regulator of the fate determination, self-renewal, and differentiation of the dormant HBCs 108, 109.

GBCs are heterogeneous in morphology and express *Lgr5* exclusively through Wnt-signaling 110. The neural differentiation potential of GBCs is coordinated by sequential and distinct but overlapping expression of neural transcription factors, including *Ascl1*, *Ngn1*, and *NeuroD1* 111. GBCs and HBCs may represent the active and reserve stem cell populations, respectively, during the homeostasis of the OE 112.

Hauser *et al.*, have identified a new type of resident stem cells called olfactory ecto-mesenchymal stem cells (OE-MSCs) in the lamina propria (LP)^113^. These cells were derived from the neural crest and showed a proliferation profile and multiple differentiation potential like bone marrow mesenchymal stem cells (BM-MSCs) 114. OE-MSCs possess immunomodulatory activity *in vitro* as MSCs from other sources 114. Finally, although the vomeronasal organ (VNO) is not a part of the OE, VNO is the secondary olfactory organ located in the lower part of the nasal cavity. It is primarily known to detect sexually and socially relevant chemicals and is thought to contain neural stem cells (NSCs) 115. In the VNO, there are two types of stem cells: one mainly maintaining the VNO and the other replacing the lost neural cells 115.

### Differentiation of olfactory stem cells *in vitro and in vivo*

HBCs are quiescent in general and don't proliferate *in vivo* and *vitro.* However, Peterson *et al.,* could culture HBCs in a medium mimicking the respiratory epithelium (RE) culture system, including dual-SMAD inhibitors and TGFα 116. Following excision of* p63* and retinoic acid treatment, HBCs can be activated and differentiated into neuronal and non-neuronal lineage cells (including GBCs), respectively 116. Moreover, engrafted HBCs fully restore the methyl bromide-lesioned OE by differentiating to all the OE cell types, including ORNs 116. Attempts have also been made to purify and expand GBCs *in vitro*. GBCs were isolated using immunoselection with antibodies against GBC-specific markers and expanded to large quantities in the presence of TGFβR1 inhibitors 117, 118.

### Stem cell therapy for COVID-19-associated persistent OD

While the transient OE loss could be restored naturally from activation and differentiation of basal stem cells, the permanent olfactory loss may incur more than 10% of COVID-19 survivors. While nasal steroid and anti-inflammatory sprays are widely used to accelerate the restoration of transient damages, stem cell-based approaches could provide a lasting solution in curing permanent OD.

First, the persistent inflammation in the OE after SARS-CoV-2 infection may attribute to persistent and permanent smell loss. MSCs have been implicated as an excellent cell type to alleviate dysregulated immunity through the secretion of anti-inflammatory cytokines and the expression of immunomodulatory surface proteins 119, 120. MSCs are easy to culture, scale up, and characterize *in vitro* and can be easily tested in animal models 121-124. Furthermore, MSCs are low in immunogenicity in the allogeneic engraftment 125, 126. Pre-conditioning of MSCs via hypoxic culture or treatment with an inflammatory stimulus such as lipopolysaccharide enhances their immunomodulatory effects due to increased secretion of anti-inflammatory cytokines, including NO, IL-10, and TGFβ, and decreased secretion of inflammatory cytokines, including IL-1, IL-6, and TNFα 127. Finally, MSCs can directly modulate T cell activity by suppressing effector T cell proliferation and increasing regulatory T cell expansion through elevated expression of immune-modulatory ligands such as programmed cell death ligand-1 123, 128. Given the presence and potential immunomodulatory effects of OE-MSCs *in vivo*, the effective engraftment of the exogenous MSCs into the nasal mucosa or OE could diminish the OE damage and functional loss of ORNs. However, the retention of locally injected MSCs into the OE may gradually go down due to wash-out, cell death, and immune rejection. Antibody-assisted or genetically modified targeting can efficiently deliver and retain the cells on-site 115. For example, SCs are the only cell types that express K18 in the OE. By labeling MSCs with a peptide or an antibody that binds to K18, local injection of the modified MSCs could maximize the retention of the cells, thus reducing the inflammatory environment and accelerating the restoration of the olfactory functions.

Second, the engraftment of HBCs and GBCs may hold a good promise for OD patients whose HBCs and GBCs are depleted due to viral infection or chronic NF-κB mediated inflammation. In multiple animal models, in which OE legion is induced via olfactory bulbectomy or toxic chemicals such as methyl bromide, engraftment of HBCs and GBCs has fully restored the OE 116, 117. Kurtenbach *et al.,* developed an inducible hyposmia mouse model via conditional deletion of *Ift88,* a ciliopathy-related gene in ORNs 117. In this model, without ciliation, ORNs are incapable of odor transduction, whereas intranasal delivery of purified GBCs can produce odor-responsive ORNs, reinnervate olfactory bulbs, and recover the olfactory behavior of injured mice 117. It has also been reported that *in vitro* cultured HBCs, following engraftment into methyl bromide-lesioned OE, can restore the OE by regenerating both neuronal and non-neuronal cell types in the OE 116.

Third, it is thought that neural stem cells (NSCs) residing in the VNO can replenish damaged olfactory bulb neurons. For such NSC-based cell therapy, it is essential to understand the differentiation potential of olfactory NSCs *in vivo*. Li *et al.,* recently showed that VNO-derived NSCs migrated to the olfactory bulb and differentiated into olfactory interneurons after engraftment into the subventricular zone 129. In another study, strips of OE were directly grafted to the olfactory bulb with long-term survival. However, it was not addressed whether the damaged OE functions were restored 130. The stem cell therapies of OD in humans and animal models are summarized in Table 1.

In addition to the great therapeutic potential, some potential risk of stem cell therapy must be cautiously considered, including immune reactivity, side effects and safety. Ethical consideration or immune rejection can be solved by using autologous derived stem cells. In the pilot study discussed above, no serious adverse effect or death directly related to the implantation of stem cells. Concerns about the biosafety of stem cell transplantation have been reported in many other studies, and the behavior of transplanted stem cells can be guaranteed in many ways 131. Nonetheless, many factors still challenge the establishment of stem cell based therapy in treating OD, such as the source, density and quality of stem cells, the administration route, dosage and frequency.

## Conclusion and perspectives

Olfactory dysfunction, the most detected symptoms in long COVID, severely affects the quality of life. While most of the smell impairments are minor in their nature and fully restored within 4 weeks, thanks to the regenerative capability of stem cells residing in the basal layer of the OE. However, more than 10% of the affected, which could be extrapolated to affect more than 40 million people worldwide, may have compromised quality of life and even face life-threatening risks due to their inability to detect toxic compounds, fire, or gas. As outlined in the review, SARS-CoV-2 may infect *ACE2*-expressing cells, including SCs, MCs, and BGDCs, in the OE and hijack the essential cellular functions, such as the maintenance of the integrity of the ORN organization and the transmission of odorant signaling through the GPCRs and neural synapses. Surprisingly, ORNs and even OBNs were infected according to animal studies and analysis of postmortem samples, although these cells do not express the receptors for the viral entry.

Since stem cells in the OE express ACE2, they can also be targeted by SARS-CoV-2. The findings thus far have strongly suggested compromised regenerative potential of stem cells in the OE contributing to permanent OD. The persistent or permanent OD is attributable to both cell-autonomous and non-cell-autonomous mechanisms in which the regenerative potential of the OE is compromised. Further underpinning the cellular and molecular mechanisms may provide a new basis for immediate and long-term translational research on various olfactory stem cells and testing their therapeutic potential.

## Figures and Tables

**Table 1:** A summary of stem cell therapies of OD used in humans and animal models.

Stem cell	Source	Species	Disease model	Administration	Function	Effect	Refs.
Mouse GBC	Olfactory epithelium	Mouse	Inducible hyposmia mouse model by conditional deletion of the IFT88 gene	Intranasally delivering the cell droplets	Grafted GBC can engraft into the OE, produce odor-responsive OSN, and reinnervate the OB	Cell treated mice show recovered olfactory behavior	117
Mouse HBC	Olfactory epithelium	Mouse	Olfactotoxic gas methyl bromide lesion	Intranasally grafting	RA pretreated HBC can engraft into lesioned OE, and generate all OE cell types, including OSN	*In vitro* cultured and expanded HBC can contribute to the regeneration of lesioned OE after transplantation	116
Mouse Neural stem cells	Olfactory epithelium	Mouse	None	Heterotopic grafting through Stereotaxic injection	OE-NSC can integrate the SVZ and proliferate, further migrate towards OB, and differentiate into neuron in the OB	OE-NSC dierived neurons exhibit electrophysilogical properties similar to endogenous neurons	129
Rat BMMSC	Cell line	Rat	Triton X-100 irrigation to injure the OM	Cell suspension locally injected into OE	Transplanted BMSC can engraft to the damaged OM and elevat the expression of nerve growth factor and brain-derived neurotrophic factor	Cell treated OM have restored cellular composition	132
Human ADSC	Aspirated adipose tissue	Mouse	Dichlobenil inoculation to damage the olfactory region	Cell suspension injected through tail vein	Transplanted ADSC can engraft in the lesioned OE and induce neuroregenerative process	Cell treated mice can respond to odorant stimulation activity through electroolfactogram test	133

## Abbreviations

COVID-19: Coronavirus disease-19
SARS-CoV-2: Severe acute respiratory syndrome coronavirus-2
OD: Olfactory dysfunction
CNS: Central nervous system
BBB: Blood-brain barrier
ACE2: Angiotensin-converting enzyme 2
TMPRSS2: Transmembrane serine protease 2
OM: Olfactory mucosa
OE: Olfactory epithelium
RE: Respiratory epithelium
LP: Lamina propria
ORN: Olfactory receptor neuron
OR: Olfactory receptor
OB: Olfactory bulb
SC: Sustentacular cell
MVC: Microvilli cell
BGDC: Bowman's gland ductal cell
HBC: Horizontal basal cell
GBC: Globose basal cell
GPCR: G protein-coupled receptor
IFN: Interferon
ISG: IFN-stimulated genes
DPI: Days post-infection
Scrams: Single-cell RNA-sequencing
BCEC: Brain capillary endothelial-like cell
OMP: Olfactory marker protein
OE-MSC: Olfactory ecto-mesenchymal stem cells
VNO: Vomeronasal Organ
OBX: Olfactory bulbectomy
NSC: Neural stem cell
IF: Immunofluorescence
ISH: *In situ* hybridization
HCC: Histocytochemistry
